# Effects of Neck-Arm Restraint Suspension of Beef Carcasses on Meat Quality and Proteome of Different Muscles During Post-mortem Aging

**DOI:** 10.3389/fnut.2021.774529

**Published:** 2021-12-21

**Authors:** Wentao Cai, Kaixin Wen, Leijie Che, Haijun Zhang, Yang Zhang, Junya Li, Haipeng Li

**Affiliations:** ^1^Institute of Animal Science, Chinese Academy of Agricultural Sciences, Beijing, China; ^2^National Genetic Resources Protection Center of Jinnan Cattle, Yuncheng, China; ^3^Xinjiang Academic of Animal Science, Urumqi, China

**Keywords:** beef quality, carcass, neck-arm restraint suspension, TMT, proteomics

## Abstract

Beef quality is the first deciding factor for consumers to consider before purchasing. The aim of this study was to evaluate the effects of suspension and aging time on beef quality. We compared the differences in pH, drip loss, cooking loss, color, shear force, myofibril fragmentation index (MFI), and electron microscope of three muscle tissues between Achilles tendon (AT) and neck-arm restraint (NR) suspensions during seven aging periods (days 0, 1, 2, 3, 7, 14, and 21) after slaughter using the carcasses of six Xinjiang brown cattle. We found that NR suspension could significantly increase the water loss rate and MFI, as well as reduce the shear force compared to AT suspension. The muscle fiber structure with NR suspension was more severely damaged. The proteomics of longissimus dorsi was checked for the post-mortem days 1, 7, and 14. We detected 50, 26, and 29 differentially expressed proteins between NR and AT suspension at post-mortem days 1, 7, and 14, respectively. These proteins were involved in metabolic and muscle structure associated pathways and contributed to a comprehensive understanding of suspension-dependent meat quality regulation by proteins in beef cattle. To conclude, NR suspension can accelerate the aging time of beef carcasses, which will reduce the cost of carcass suspension and bring more benefits in the beef industry.

## Introduction

Meat is the important nutrient-intensive food that has been worldwide consumed. Meat quality is affected by many factors, such as tenderness, flavor, and juiciness (1). Tenderness is the main quality attribute of beef and the main reason for consumers' willingness to repurchase and acceptability (2). Tenderness is dependent upon animal's age, breed, sex, nutritional status, post-mortem aging, manner of suspension, and other factors. In beef industry, the suspension is often considered to be one of the important factors determining the ultimate tenderness of the meat (3).

There was a linkage between improved tenderness and sarcomere length increases (4). The carcass suspended method had a major influence on sarcomere length and tenderness (5). It has been reported that hanging carcasses using different configurations in the cooler increased tenderness by stretching certain muscles and decreased tenderness by relaxing others (6). Achilles tendon (AT) suspension, pulling the hind leg backward in a position unlike the normal muscle configuration of a standing animal, is a conventional method for carcasses hanging during the slaughter process. However, this method brings less skeletal restraint on a large proportion muscle of the hind limb. By AT suspension, the vertebral column is less stretched and more curved, and pushed together, which allows more muscle shortening for the longissimus dorsi (LD) during the rigor process (7).

Pelvis suspension is a carcass hanging configuration, in which the carcasses are hanged by pelvic bone. Pelvis suspension increased tension over loin and hindquarter muscles during rigor establishment, avoided intense contraction, and turned them more tender compared to AT suspension (8). For beef carcasses, stretched muscle has been proved to be tender than corresponding ones under reduced tension (9). Nevertheless, the hind leg hanging in a 90° position might require additional space for the carcasses or sides in chilling rooms (3). Tendercut technology is another tenderness intervention that involves cutting through bones without damaging the major muscles, which are more stretched and tender for muscles (10). However, the tendercut requires more work, and the round/sirloin cut seems to be dependent on well-defined criteria for the specific cutting (3).

Although the toughness of beef is a critical problem that can be solved by long time aging (14–21 days), a lot of energy may be wasted by extended aging. Neck-arm restraint (NR) suspended technique is a suspension imposing a radial external force between the distal end of the forearm and the front end of the neck. The NR suspension can keep the LD stretch, which may prevent the contraction of LD and forelimb muscles. However, little information is available about the effects of NR suspension on beef quality. It is uncertain whether beef with NR suspension can achieve higher tenderness compared with AT suspension.

The rising quantitative proteomic technology allows the systematic study of proteome expression profiles and the identification of meat quality biomarkers (11, 12). Recently, several studies have been carried out to elucidate muscle development and meat quality traits in cattle through proteomics-based methods (12). However, there are still limited studies examining the changes in protein solubility with different suspended methods for beef cattle during post-mortem aging.

Neck-arm restraint suspension can increase the tension of loin and hindquarter compared to AT suspension. Hence, our hypothesis is that hanging carcasses by NR method may have an impact on the tenderness of some muscles. In this way, the objectives of this study were to evaluate the effects of NR suspension on the beef quality (pH, drip loss, cooking loss, shear force, color difference, and myofibril fragmentation index [MFI]) during post-mortem days 0, 1, 2, 3, 7, 14, and 21. The protein expression profiles of LD and related biomarkers contributing to meat quality were also investigated under different suspension methods.

## Materials and Methods

### Animals and Samples Collection

The Xinjiang brown cattle is an important breed in the beef industry of Xinjiang Uygur Autonomous Region, China. In this study, six Xinjiang brown bulls with ~30 months of age and similar body masses were slaughtered at a local slaughterhouse (Yining, Xinjiang, China) according to the commercial procedures. All corn-fed calves were raised on the same farm to ensure consistent background and then slaughtered using electrical stunning on the same day. After slaughter, for each cattle, the right half of carcass was matured with the traditional AT suspension. The left half of carcass was matured by NR suspension, which used nylon rope to tie the distal end of forearm to the junction of the first and second cervical vertebrae and continued to apply a radial pulling force of 10–50 kg. The carcasses were overhung in a cold room (3 ± 1°C) with 85–90% humidity for 21 days. Then, the biceps femoris (BF), LD, and triceps brachii (TB) of the right and left half of carcasses were collected for each carcass at days 0, 1, 2, 3, 7, 14, and 21 after slaughter. All procedures were undertaken the guidelines given by the Animal Care and Ethics Committee for animal experiments, Institute of Animal Science, Chinese Academy of Agricultural Sciences.

### Beef Quality Measurements

For assessing the beef quality, we measured the pH, drip loss, cooking loss, shear force, and color difference. The pH value of the beef was measured at the post-mortem days 0, 1, 2, 3, 7, 14, and 21 following the method described by the previous study with minor modifications (13). Chopped meat (10 g) was mixed with 100 ml of distilled water for 15 s, and then homogenized using an Ultra-Turrax T25 homogenizer at 2,800 *g*. The pH of the homogenate was measured using a pH meter equipped with an electrode (PB-10, the precision was 0.01). The drip loss was measured by Strain controlled unconfined pressure gauge (yyw-2, Nanjing Soil Instrument Factory Co., Ltd, China). For the cooking loss, the beef was weighed as *m*_1_ after removing the fascia and fat, and then was heated in an Electro-Thermostatic Water Bath (Dk-s28, Qingdao Mingbo Environmental Protection Technology Co., Ltd, China) at 80°C, when the central temperature of the beef reached 70°C, the beef was weighed as *m*_2_, the cooking loss rate (%) was calculated according to the formula:


$$\begin{array}{l} \text{cooking loss rate }\left(\text{\%}\right)=\frac{\text{m}1-\text{m}2}{\text{m}1}\times \;100\text{\%}. \end{array}$$

The shear force measurement was performed according to the determination method of national standard (NY/T 1180-2006) with the Texture analyzer (TA-XT2i, SMS, UK). The meat color traits, L^*^, a^*^, and b^*^ values, were measured by the Colorimeter (CR-400, Beijing kermeirunda Instrument Equipment Co., Ltd., China). In this study, all the measured values were performed in triplicate, and the average statistics were as the normalized results.

### Beef Histologic Evaluation

To investigate the MFI of beef, 2 g beef was weighted after removing the visible fat, and co-centrifuged with the MFI buffer including 100 mmol/L KCl, 11.2 mmol/L K_2_HPO_4_, 8.8 mmol/L KH_2_PO_4_, 1 mmol/L ethylene glycol diethyl ether diamine tetra acetic acid, 1 mmol/L MgCl_2_, and 1 mmol/L NaN_3_. Further, 5 ml MFI buffer was added into the precipitation after discarding the supernatant, and then the connective tissue was filtered out to obtain the myofibrillar protein suspension. The protein content of myofibrillar protein suspension was measured by the biuret method, and was adjusted to 0.5 mg/ml through MFI buffer (14). The optical density (OD) was measured at 540 nm by using spectrophotometer. The MFI was obtained by multiplying OD with 200. For each sample, the MFI values were performed in triplicate, and the average statistic was as the normalized result. In addition, the meat samples were cut into cuboids of 3 × 1 × 1 mm, fixed with glutaraldehyde solution, washed with phosphoric acid buffer, dehydrated by ethanol gradient, and replaced by anhydrous acetone. After embedding, slicing, and staining the meat samples, we observed and photographed using the transmission electron microscope (H-7500, Hitachi Limited, Tokyo, Japan).

### Protein Extraction

The 36 LD samples were collected from six AT suspension right carcasses and six NR suspension left carcasses at post-mortem days 1, 7, and 14. After grinding the tissues by liquid nitrogen, two samples were randomly selected for pooling together in each suspended group, which generated 18 samples for six groups and each group contained three biological replications. Protein was extracted as follows: (1) Each sample was homogenized in extraction sodium dodecyl sulfate (SDS) buffer such as 4% SDS, 1 mM dithiothreitol, and 150 mM Tris-HCl (pH 8.0). (2) The homogenate was sonicated 2 min after 5 min incubation in boiling water. (3) The crude extract was incubated in boiling water 5 min again and then clarified by centrifugation at 20,000 *g* for 10 min at 25°C. (4) The concentrations of proteins were quantified by BCA protein assay reagent (Shanghai Bioprofile Technology, Shanghai, China). (5) The integrities of samples were measured with electrophoresis on 8–16% SDS-polyacrylamide gels and stained with Coomassie Brilliant Blue.

### Protein Digestion and Tandem Mass Tags Labeling

For the protein digestion, 300 μg of protein isolated from each sample was mixed with dithiothreitol to 100 mM. After 5 min incubation in boiling water, the protein solution was cooled to room temperature, and then was mixed with 200 μL UA buffer (8 M Urea, 150 mM Tris-HCl, pH 8.0) to centrifuge at 12,000 *g* for 15 min. After discarding the filtrate, 100 μL IAA (50 mM IAA in UA) was added into the sediment, and the mixture was vibrated for 1 min, kept away from light for 30 min, and centrifuged for 10 min at 12,000 *g*. Processes adding 100 μL UA buffer and centrifuging the mixture for 10 min at 12,000 *g* were performed twice. And then, processes adding 100 μL NH_4_HCO_3_ buffer and centrifuging the mixture for 10 min at 14,000 *g* were also performed twice. Finally, 40 μL trypsin buffer (6 μg Trypsin in 40 μL NH_4_HCO_3_ buffer) was added into the precipitate to obtain the filtrate *via* vibrating for 1 min, keeping 37°C for 16–18 h, and centrifugation for 10 min at 12,000 *g*. The peptide was quantified by the C18 cartridge desalination.

Peptide mixture from each filter was labeled using tandem mass tags (TMT) reagents (Thermo Fisher, MA, USA) to quantify simultaneously up to 18 samples. For each TMT set, six isobaric compounds were used to label different samples of the study group according to the manufacturer's instructions. Briefly, the labeled peptides of each group were mixed equally, and separated by the High-pH (Pierce™ High pH Reversed-Phase Peptide Fractionation kit, Thermo Fisher, MA, USA) after drying the peptides. Finally, a total of 45 components were obtained by three labeling, and the peptides of each component were dried and redissolved with 0.1% FA.

### Data Analysis

Maxquant software (v1.6.0.16) was used to identify and quantify the peptide against UniPort Proteomes-*Bos taurus*-46707-20190911.fasta with the following parameters: type ~ reporter ion MS2, isobaric labels ~ TMT 6plex, enzyme ~ trypsin, reporter mass tolerance ~ 0.005 Da, max missed cleavages ~ 2, main search peptide tolerance ~ 4.5 ppm, first search peptide tolerance ~ 20 ppm, MS/MS tolerance ~ 20 ppm, fixed modifications ~ carbamidomethyl, variable modifications ~ oxidation and acetyl, database pattern ~ target-reverse, PSM-FDR ~ < 0.01, and protein FDR ~ < 0.01. The proteins were quantified by razor and unique peptides. The differentially expressed protein (DEP) analysis was performed with one-way ANOVA using ANOVA function of R Stats Package (version 3.4.3). The proteins with *P*-value < 0.05 and fold change > 1.2 or < 0.83 were considered as DEPs.

### Biological Function Analysis

The Kyoto encyclopedia of genes and genomes (KEGG) pathways and functional enrichment of gene ontology (GO) terms were performed for DEPs in the KOBAS database (http://kobas.cbi.pku.edu.cn/kobas3/genelist/) (15). Both KEGG pathways and GO terms with the *P*-values < 0.05 were considered to be significantly enriched. In addition, the protein–protein interaction (PPI) networks were conducted with the String database and Cytoscape software (16, 17).

### Parallel Reaction Monitoring Validation

To further validate the protein expression level gained through TMT quantification, additional quantification by parallel reaction monitoring (PRM) analysis was performed. Protein extraction, trypsin digestion, and LC–MS/MS analysis were performed as described previously (18). The raw data were analyzed using Skyline 4.1 to obtain the signal intensities of individual peptide sequences (19). For the PRM–MS data, each sample's average base peak intensity was extracted from the full scan acquisition using RawMeat (version 2.1, VAST Scientific). The normalization factor for sample N was calculated as f_*N*_ = the average base peak intensity of sample *N* divided by the median of the average base peak intensities of all samples. The area under the curve (AUC) for each transition from sample N was multiplied by this factor. After normalization, the AUC of each transition was summed to obtain AUCs at the peptide level. The relative protein abundance was defined as the intensity of a certain peptide.

### Statistics

Statistical analysis was conducted by SPSS 20.0 using the independent *t*-test. Differences with *P*-value < 0.05 were considered to be significant. Results are shown as the means and standard errors.

## Results

### Measurement of Beef Quality

**Figure 1:**
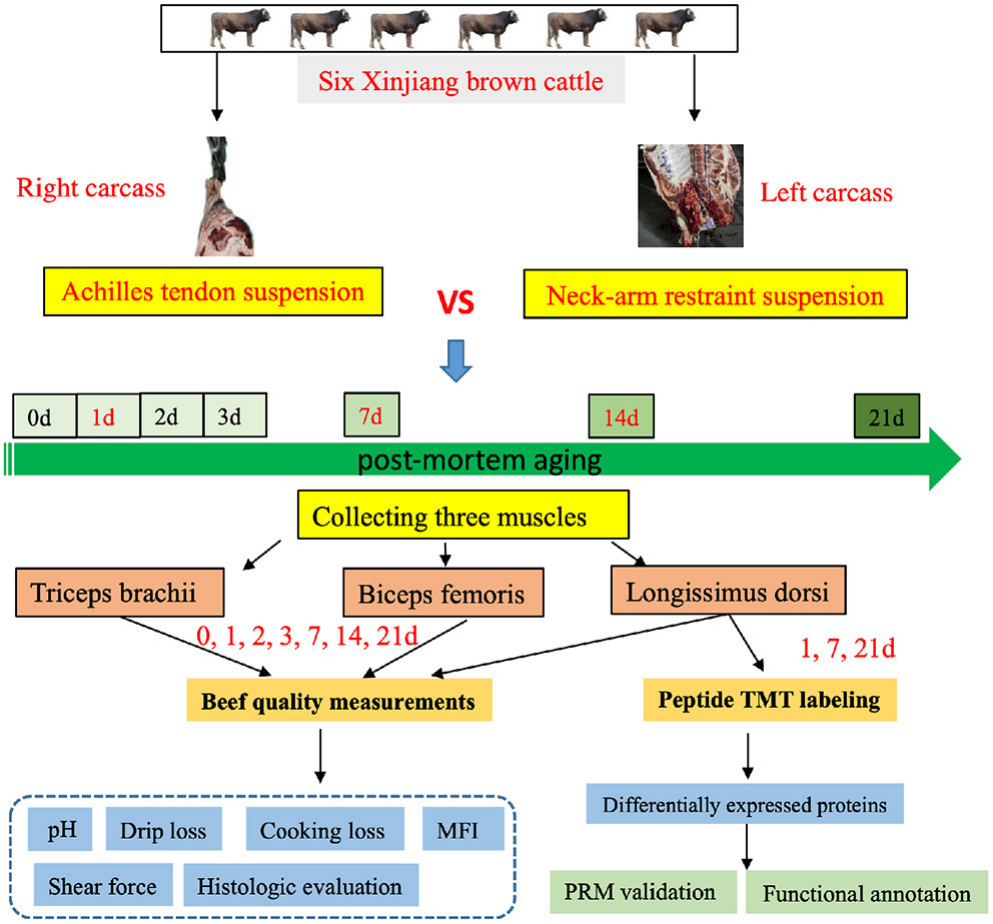
The experimental design of this study. Flowchart shows detailing the experiments such as the sample preparation, quality measurements, and data analysis.

Our experimental workflow is shown in Figure 1. We measured the pH, drip loss, cooking loss, shear force, color difference, and microstructure for right half of carcass matured with AT suspension and left half of carcass matured with NR suspension in BF, LD, and TB from six Xinjiang brown cattle at days 1, 7, and 14 after slaughter.

#### Determination of pH

**Figure 2:**
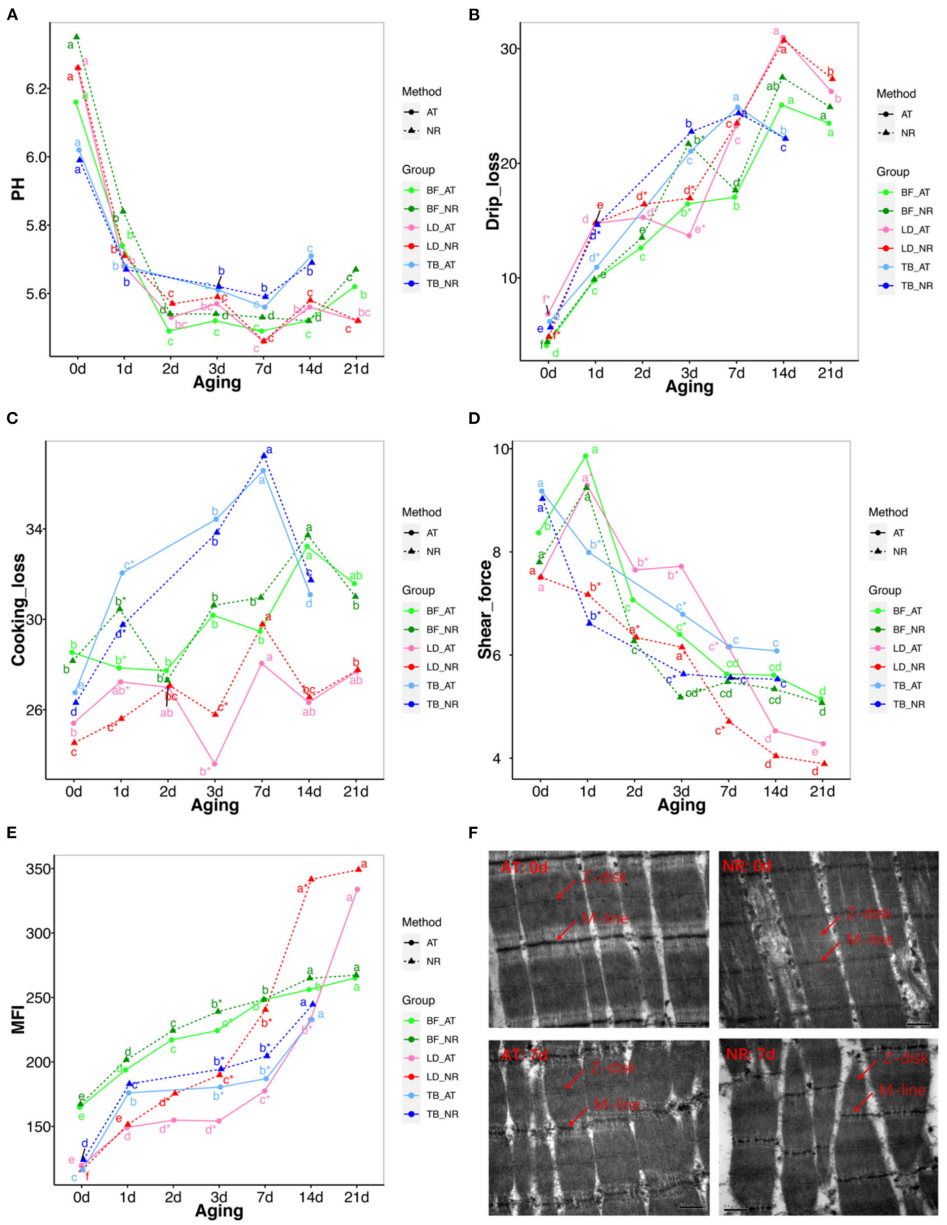
Changes in pH, drips loss, cooking loss, shear force, myofibril fragmentation index (MFI) and muscle microstructure between achilles tendon (AT) and neck-arm restraint (NR) in biceps femoris (BF), longissimus dorsi (LD), and triceps brachii (TB) muscles during aging time. **(A–E)** Changes in pH, drips loss, cooking loss, shear force, MFI between AT and NR in BF, LD, and TB muscles from days 1 to 21 after slaughter. The lines with green, red, and blue represent BF, LD, and TB, respectively. The solid and dashed line represent AT carcasses suspension and NR carcasses suspension, respectively. *Symbol means the values are significantly different between AT and NR suspension (*P*-value < 0.05). a–g means the values are significantly changed during aging (*P*-value < 0.05). **(F)** Transmission electron microscopy (TEM) images of LD at post-mortem days 0 and 7 aging periods. The up-left and up-right of pictures represent the LD at post-mortem day 0 with AT suspension and NR suspension, respectively. The down-left and down-right of pictures represent the LD at post-mortem day 7 with AT suspension and NR suspension, respectively. The Z-disc and M-line information were marked in these pictures.

A significant decrease of pH value could be seen during the first 72 h storage in both suspended groups. There was no considerable pH difference between the two suspended methods during aging (Figure 2A; Table S1A).

#### Drip Loss and Cooking Loss Analysis

The increase trend of drip loss was found in both NR and AT suspended groups during aging time. The drip loss of BF and TB with NR suspension were significantly increased compared with AT suspension at post-mortem day 3 (*P* = 0.013) and 1 (*P* = 0.036), respectively. The LD showed significantly higher drip loss with NR suspension than those with AT suspension at post-mortem day 2 (*P* = 0.011) and 3 (*P* = 0.009) (Figure 2B; Table S1B). In addition, the cooking loss of LD and TB with NR suspension was significantly lower than that with AT suspension at post-mortem day 1 (Figure 2C; Table S1C).

#### Quality Classification of Meat Color

The results of measured meat color were shown in Figure S1; Table S1D. The L^*^, a^*^, and b^*^ values increased in both NR and AT suspensions with the growing aging time. The L^*^ of LD significantly decreased with NR suspension at post-mortem day 1 (*P* = 0.017) and 14 (*P* = 0.036), and the a^*^ of LD significantly decreased with NR suspension at post-mortem day 1 (*P* = 0.023), 7 (*P* = 0.029), and 14 (*P* = 0.047), while the b^*^ of LD significantly increased with NR suspension at post-mortem day 14 (*P* = 0.033). The L^*^ and a^*^ values at other aging time of NR suspension were lower than those of AT suspension, these differences were not statistically significant. The difference of TB and BF between suspension methods was limited. We did not see the significant changes in L^*^ between two suspension methods in both TB and BF during aging. The a^*^ and b^*^ values of BF with NR suspension decreased at post-mortem day 1 and 21, while the a^*^ and b^*^ values of TB with NR suspension increased at post-mortem days 1 and 7, respectively.

#### Assignment of Shear Force

The effects of suspended methods and aging time on shear force are presented in Figure 2D; Table S1E. Both suspended method and aging time affected the shear force of BF, LD, and TB. The decrement of shear force was found in NR and AT suspended groups during aging time. For BF, the significant decrease tendency of NR suspension only was found at post-mortem day 3 (*P* = 0.007). The shear force of LD decreased significantly after NR suspension compared with AT during post-mortem day 1 to day 7 (*P* < 0.05). For TB, the shear force of NR suspension was significantly decreased at post-mortem day 1 (*P* = 0.033) and 3 (*P* = 0.012). The shear force of NR suspension at other post-mortem aging was also lower than that of AT suspension, while these differences were not statistically significant. We found the tenderness of NR suspension at post-mortem day 3 was similar with that of AT suspension at post-mortem day 7. In addition, the average gaps of shear force between NR and AT from post-mortem days 1–14 were 0.612, 1.388, and 0.768 kg for BF, LD, and TB, respectively.

#### Measurement of Myofibril Fragmentation Index

MFI increased in both NR and AT suspended methods from days 1 to 21 after slaughter (Figure 2E; Table S1F). For BF, the MFI values of NR suspension only increased at post-mortem day 3 compared with the AT method (*P* = 0.009). The MFI values of LD matured with NR significantly increased at post-mortem day 2 (*P* = 0.003), 3 (*P* = 0.003), 7 (*P* = 0.001), and 14 (*P* = 0.002). The MFI values of TB matured with NR suspension were larger at post-mortem days 3 and 7 than those with AT suspension. Additionally, the average increases of MFI during post-mortem days 1–14 were 7.788 for BF, 46.076 for LD, and 12.565 for TB.

#### Muscle Microstructure

We performed the muscle microstructure and found that the sarcoplasmic reticulum around the sarcomeres can be clearly distinguished and the myofibrils are tightly combined with the visible I-band and A-band and the Z-disk and M-line can be differentiated at post-mortem day 0. After 7 days, the overall integrity of the myofibrils diminishes. The worst myofibrillar structure is observed at NR suspended group in which the overlapping structure of thick and thin filaments was destroyed, the skeletal muscle structure was severely broken, and the Z-disk was distorted and weakened (Figure 2F).

### Identification and Quantification of Protein

**Figure 3:**
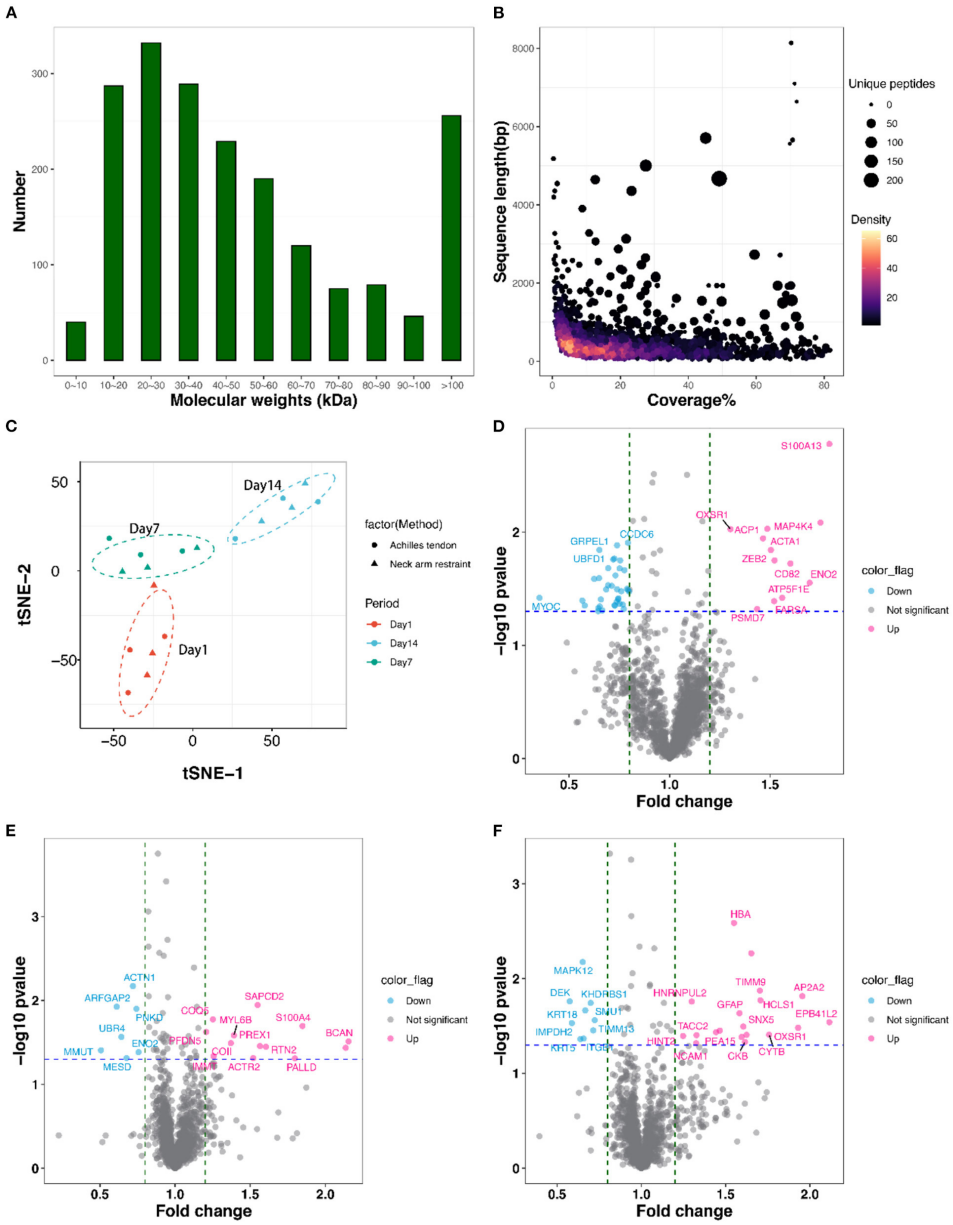
Features of the identified proteins. **(A)** The mass distribution of all identified proteins. **(B)** The coverage, sequence length, and unique peptides number of all identified proteins. **(C)** Clustering analysis of all 18 proteomic samples using *t*-Distributed Stochastic Neighbor Embedding (*t*-SNE) procedure. **(D–F)** Volcano plots display differentially expressed proteins (DEPs) between AT and NR suspension at post-mortem days 1, 7, and 14. The hot pink dots represent the up-regulated DEPs; the light blue dots represent the down-regulated DEPs.

We conducted the proteome of 18 LD samples with NR and AT suspensions across three post-mortem stages of 1, 7, and 14 d by TMT. In total, we obtained 12,900 peptide spectrum matching numbers and 12,056 unique peptides, which were mapped to 1,943 proteins with the FDR <1%. More than 68% of the identified proteins had molecular weights in the range of 10–20 kD (287), 20–30 kD (332), 30–40 kD (289), 40–50 kD (229), and 50–60 kD (190) (Figure 3A). Approximately 70% of the identified proteins had at least three unique peptides. Additionally, the identified proteins had high peptide coverage, of which, 40 and 65% proteins showed more than 20 and 10% sequence coverage, respectively (Figure 3B). Details of all the identified proteins are shown in Table S2. Despite differences in sample characteristics, samples from the same aging of suspensions clustered together based on their protein expression profiles (Figure 3C).

### Differentially Expressed Proteins of Comparison Groups

**Figure 4:**
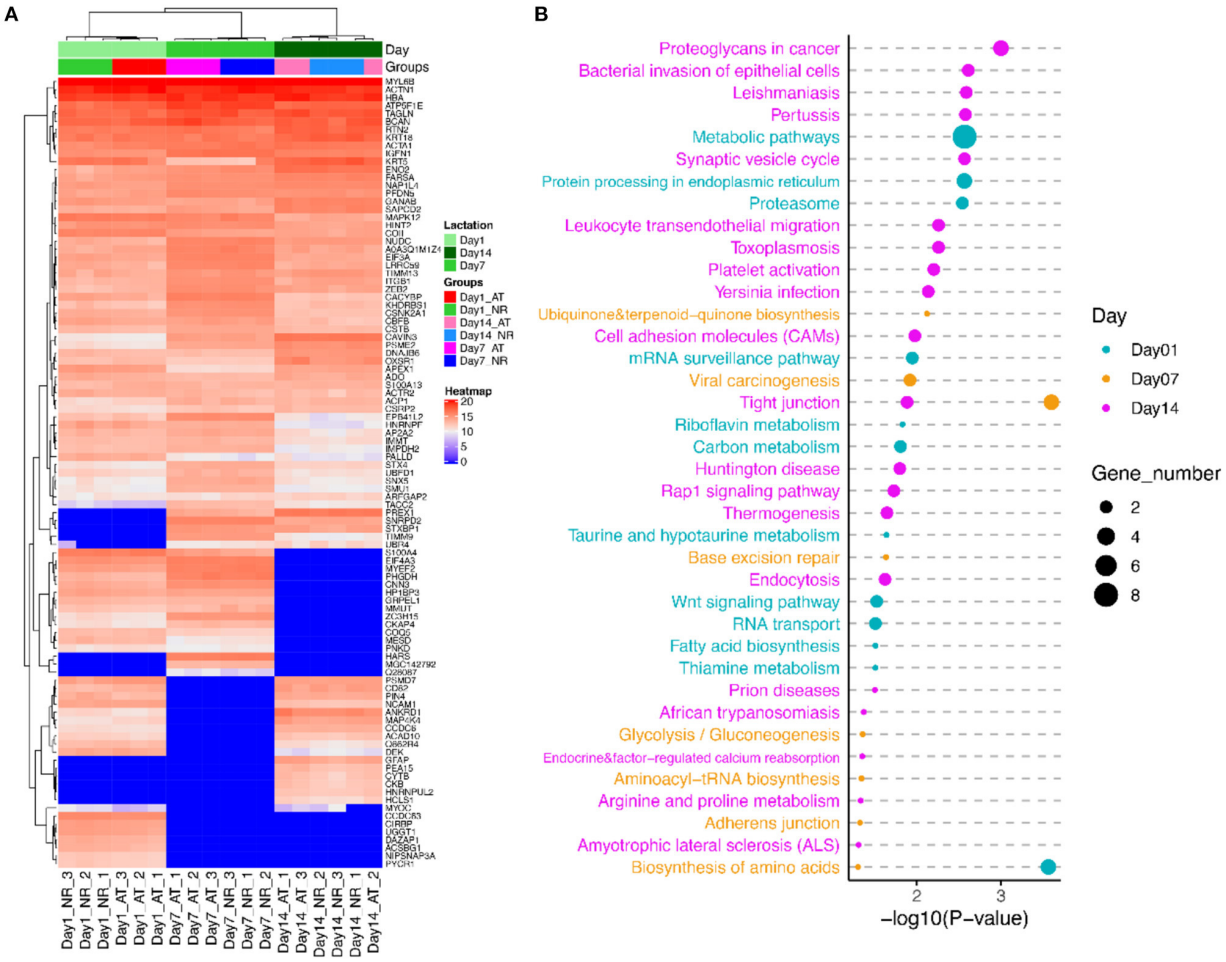
The cluster and pathway analysis of DEPs of AT and NR suspension at post-mortem days 1, 7, and 14. **(A)** Cluster analysis of DEPs based on their expression. Red indicates higher expression, and blue indicates lower expression. **(B)** Predominant function categories targeted by DEPs at post-mortem days 1, 7, and 14. Larger significant values and shapes suggest higher relevance and higher enriched fold, respectively.

As shown in the volcano plots (Figures 3D–F), we identified 92 DEPs with *P*-value < 0.05 and fold change > 1.2 or < 0.83 in NR suspension vs. AT suspension using one-way ANOVA, such as 50 DEPs (12 up-regulated and 38 down-regulated), 24 DEPs (13 up-regulated and 11 down-regulated), and 29 DEPs (19 up-regulated and 10 down-regulated) at post-mortem days 1, 7, and 14, respectively (Table 1 and Table S3). Further, hierarchical cluster analysis of DEPs was performed to better visualize the differences in protein abundance among comparison groups, and these results were visualized as a heat map (Figure 4A). A total of 1,456, 1,352, and 1,339 DEPs were detected in 1 vs. 7 d, 7 vs. 14 d, and 1 vs. 14 d, respectively. Interestingly, we found 707 DEPs exhibited common changes across all three comparison groups (Table S4).

**Table 1:** The differentially expressed proteins (DEPs) between achilles tendon (AT) and neck-arm restraint (NR) suspension at days 1, 7, and 14 after slaughtering.

**Post-mortem aging**	**DEPs**	**Up-regulated**	**Down-regulated**
Day 1	50	12	38
Day 7	24	13	11
Day 14	29	19	10

### Functional Annotation of DEPs

To investigate the molecular mechanism of DEPs in determination of meat quality, we performed GO and KEGG enrichment analysis using KOBAS3.0. KEGG analysis showed that these DEPs at post-mortem day 1 were significantly enriched in 12 KEGG pathways, such as biosynthesis of amino acids, metabolic pathways, protein processing in endoplasmic reticulum and proteasome. GO annotation demonstrated these DEPs at post-mortem day 1 were involved in 144 GO terms, such as Z disc, actinin binding, myosin light chain binding, titin binding, I band, and fibronectin binding (Figure 4B; Table S5). The DEPs at post-mortem day 7 were associated with tight junction, viral carcinogenesis, base excision repair, adherens junction, and biosynthesis of amino acids. Their GO terms were mainly involved in cytosol, stress fiber, protein folding, Z disc, lamellipodium, and focal adhesion (Figure 4B; Table S5). The DEPs at post-mortem day 14 were enriched in 20 pathways, such as synaptic vesicle cycle, toxoplasmosis, platelet activation, cell adhesion molecules (CAMs), tight junction, and arginine and proline metabolism. GO enrichment analysis confirmed that these DEPs genes were involved in cytosol, protein binding, keratin filament, contractile fiber, actin filament binding, intermediate filament cytoskeleton organization, fibronectin binding, and cell–cell adhesion mediator activity (Figure 4B; Table S5).

**Figure 5:**
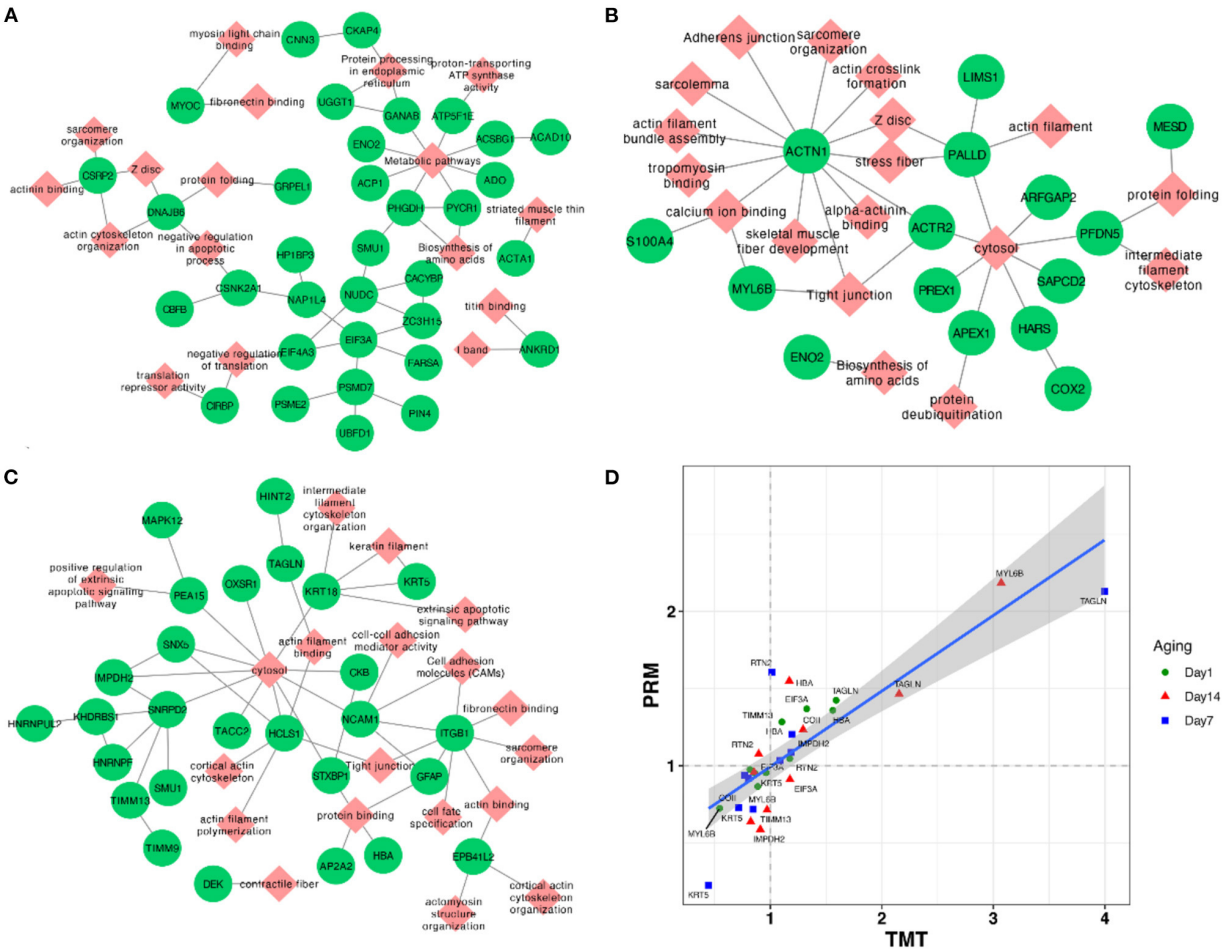
**(A–C)** Network plots of DEPs between AT and NR groups at post-mortem days 1, 7, and 14. The red diamonds and green circles represent DEPs and pathways, respectively. **(D)** The correlation plot between TMT and parallel reaction monitoring methods based on their fold change values of AT suspension vs. NR suspension.

In addition, we conducted the PPI network analysis, which showed the interaction networks of some important DEPs in different comparison groups. The PPI network analysis focused on several significantly key pathways of energy metabolism and muscle development between AT and NR at post-mortem days 1, 7, and 14 (Figures 5A–C). The results of DEPs at post-mortem day 1 showed that MYOC (Myocilin) was enriched in myosin light chain binding and fibronectin binding, while CKAP4 (Cytoskeleton Associated Protein 4) and CNN3 (Calponin 3) were involved in protein processing in endoplasmic reticulum, and ATAC1 (Actin Alpha 1) was derived from striated muscle thin filament. ATP5F1E (ATP Synthase Peripheral Stalk-Membrane Subunit B) was observed to closely associate with metabolism pathways and proton-transporting ATP synthase activity (Figure 5A). The results of DEPs at post-mortem day 7 days showed that ACTN1 (Actinin Alpha 1) involved in a variety of muscle metabolism pathways or GO terms, such as Z disc, skeletal muscle fiber development, and stress fiber, while MYL6B (myosin light chain 6B) was associated with tight junction and calcium ion binding (Figure 5B). The results of DEPs at post-mortem day 14 showed that ITGB1 (Integrin Beta 1) was involved in cell adhesion modules, fibronectin binding and tight junction, and KRT5 (Keratin 5) was derived from keratin filament (Figure 5C).

### Validation of DEPs by PRM

To assess the validity of TMT analysis, we randomly selected 10 DEPs (TIMM13, COII, EIF3A, RTN2, HBA, MYL6B, IMPDH2, KRT5, TAGLN, and RPN1) to perform the PRM–MS analysis. As shown in Figure 5D, these 10 protein expression levels obtained by TMT were further confirmed through PRM–MS analysis. The Pearson correlation coefficient between the results of TMT and PRM was *R*^2^ = 0.839. The PRM results had a good correlation with the TMT data, indicating that these quantitative results were strongly convincing.

## Discussion

### Muscle Characteristics

In this study, we compared the pH, drip loss, cooking loss, color, shear force, MFI, and microstructure of BF, LD, and TB muscles between AT and NR suspended methods at days 0, 1, 2, 3, 7, 14, and 21 after slaughter. There were differences between the two suspended methods in the dimensions of BF, LD, and TB muscles. The NR suspension had no significant effect on pH, which is in agreement with pelvic suspension that did not affect the pH values (20). Drip loss of NR suspended treatment was increased in early aging for three muscles, while these increases were slightly reduced with the suspended time going. The cooking loss of NR suspended treatment was significantly reduced in LD and TB with a similar tendency in BF. These data on liquid retention suggest a structural change within the two suspended as a consequence of being stretched. This improved water holding capacity can be attributed to the less overlap between thick and thin filaments which favors expansion of the myofibrillar lattice in the presence of brine and hence larger interfilament space to hold water (21).

L^*^, a^*^, and b^*^ were increased during the aging time, which have been reported in AT and pelvic suspensions in the previous study (22). Meat color depends on the heme iron content of the meat (23). The reduction of L^*^ and b^*^ in LD with NR suspension implied that NR suspension method may affect the heme iron content of LD.

The shear force of BF, LD, and TB was decreased in NR suspended group, implying that NR suspended method could rapidly improve tenderness during post-mortem aging compared with AT suspension. The tenderness of NR suspension at post-mortem day 3 was similar with that of AT suspension at post-mortem day 7, which indicated that NR suspension could accelerate tenderization. The average increase of shear force values of LD was higher than that of TB and BF during days 1–14, suggesting NR suspended method could better improve the tenderness of LD than TB and BF.

The MFI of muscle is an important indicator of the myofibrillar structural protein degradation extent of muscle during post-mortem time (24). Here, the post-mortem day showed a significant effect on MFI with each of suspended method, which was consistent with the previous studies (22, 25). Comparing with AT suspension, the MFI of BF, LD, and TB was increased with NR suspension, implying NR suspended method accelerated degradation of the myofibrillar structural protein during post-mortem time. The decreased integrity of sarcomeres indicated the stretched muscles with NR suspended method could accelerate the fragmentation of LD.

It has been reported that the stretched muscles with longer sarcomeres and smaller fiber diameter, were also lower shear force (26). The NR suspension could keep LD stretch, which might explain why NR suspension could more obviously affect LD than TB and BF. The effect of NR treatment at post-mortem days 14 and 21 was limited that might be due to these samples became relatively tender at the later time, and had little room for further improvement.

### Proteomic Analysis

In the present study, the decrease of shear force values observed in LD with NR suspension is supported by the increase of MFI, suggesting differences in proteolytic potential among LD could exist. Therefore, a detailed investigation of the biochemical processes and protein changes in characterizing the beef tenderization may improve our understanding of the muscle related-variation in tenderness. Proteomic analysis is a powerful technique for studying the protein expression patterns, and has been widely carried out in identifying proteome changes of skeletal muscle in beef cattle (27). Here, we performed a TMT–MS/MS-based proteomic analysis of muscle at post-mortem days 1, 7, and 14, and a total of 95 DEPs were identified (FC > 1.2 or < 0.83 and *P*-value < 0.05) in NR suspension vs. AT suspension. These results strengthen our knowledge of protein temporal expression profile and make the complements to previous findings. Some discussion on the key proteins at post-mortem days 1, 7, and 14, and their association with meat quality is provided as below.

The DEPs at post-mortem day 1 were mainly associated with biosynthesis of amino acids, metabolic pathways, protein processing in endoplasmic reticulum and proteasome, Z disc, actinin binding, myosin light chain binding, titin binding, I band, and fibronectin binding. ATP5F1E, a mitochondrial membrane ATP synthase, is mainly involved in metabolic pathways and oxidative phosphorylation pathways. The previous study has reported that expression of ATP5F1E was down-regulated in tender beef animals (28). ATP5F1E was down-regulated in NR suspended group and dynamic changed between post-mortem 1 and 7 days, implying NR suspension might improve the tenderness of beef. CKAP4 is a cytoskeleton-related protein that is mainly involved in endoplasmic reticulum-related pathways. With cells gradually lose their activity after slaughter, and the protein processing in the endoplasmic reticulum gradually stagnates, which resulting in the reduction of protein content of CKAP4. ACTA1 is actin filaments, affecting muscle contraction strength and cell apoptosis. ACTA1 undergo enzymatic proteolysis during the post-mortem period. The degradation of these myofibrillar proteins is related to tenderness, and a greater occurrence of fragments than intact structures has been observed in more tender meats (29, 30). CNN3 is calmodulin 3, which is implicated in the regulation and modulation of smooth muscle contraction. CNN3 is capable of binding to actin, calmodulin, and tropomyosin. The interaction of calponin with actin inhibits the actomyosin Mg-ATPase activity. CACYBP is a class of calmodulin, which is involved in the Wnt signaling pathway, calcium-dependent ubiquitination, and subsequent degradation of target proteins by the proteasome. In early stage of post-mortem maturity, the DEPs involved in a variety of pathways, indicating various intracellular activities have not completely stopped. The NR suspension makes the skeleton protein degrade faster, which lead to a down-regulated trend in skeleton proteins. ACTA1, CNN3, and CACYBP were dynamically changed among three post-mortem aging time, which implied these three proteins could be acted as stable biomarkers during post-mortem aging. MYOC, a glycoprotein secreted by myosin, involved in cell adhesion, cell matrix adhesion, cytoskeletal organization, and cell migration. MYOC stimulates the formation of stress fibers and regulates the actin cytoskeleton synthesis through interaction with Wnt signaling pathway (31). As the dissolving of protein in muscle, the content of myosin decreases, resulting in a decrease in the content of glycoprotein. MYOC was down-regulated in NR suspended group, which implied NR suspension could stimulate the protein dissolved process.

The DEPs at post-mortem day 7 were associated with tight junction, viral carcinogenesis, base excision repair, adherens junction, biosynthesis of amino acids, cytosol, stress fiber, protein folding, Z disc, lamellipodium, and focal adhesion. COX (Cytochrome c oxidase) plays a prominent role in energy metabolism and other related processes (32). COXII (Cytochrome c oxidase subunit 2), a component of COX, received much attentions for its significant roles in apoptosis induction, pathological processes, and diseases (33). Apoptotic enzymes are related to the degradation of skeletal proteins and have an impact on tenderness of beef (34). The extensive degradation of mitochondria in carcass with NR suspension may accelerate the apoptotic process, which lead to the increase tenderness of carcass. Myosin comprises a family of ATP-dependent motor proteins that are involved in a wide range of motility processes. MYL6B regulates the light chain and relates to the vascular smooth muscle contraction. MYL6B protein identified in the present study was associated with the tenderness phenotype (29, 35). ACTN1 protein, also differentially expressed between the post-mortem days 1 and 14, was closely associated with muscle development, growth, and degradation (36). ACTN1 was located in quantitative trait loci for shear force and tenderness score in cattle (37).

The DEPs at post-mortem day 14 were enriched in CAMs, tight junction, and arginine and proline metabolism, cytosol, protein binding, keratin filament, contractile fiber, actin filament binding, intermediate filament cytoskeleton organization, fibronectin binding, and cell–cell adhesion mediator activity. ITGB1 was mainly involved in the regulation of actin cytoskeleton, tight junctions, platelet activation and ECM–receptor interaction metabolic pathways. ITGB1 was associated with cooking loss and drip loss in swine (38, 39). NCAM1 (Neural CAM 1) encodes a cell adhesion protein which is a member of the immunoglobulin superfamily. NCAM1 has been reported to be associated with meat quality traits in swine (40). Both of ITGB1 and NCAM1 proteins were dynamically changed between AT and NR suspension, implying NR suspension can help improve the meat quality. The cytoskeleton protein showed a gradual decrease in the early stage of maturation. However, the cytoskeleton protein was no longer detected, instead the non-degradable keratin (KRT5) was left at the late stage of maturity 14 days, indicating the degradation of the skeleton fibrin can affect the tenderization of muscle, and the effect becomes smaller in the late stage of maturity. These three proteins were also shown significant changes among post-mortem 1, 7, and 14 days.

## Conclusion

In this study, it can be concluded that the meat characteristics of BF, LD, and TB responded differently to the storage time and were significantly affected by the suspended method. The meat quality parameters of LD, BF, and TB were significantly improved using NR suspension, which was due to its higher water loss rate and MFI and lower shear force. The temporal expression patterns of some suspension-dependent proteins were determined and elucidated the dynamic changes in the LD muscle proteomes with the suspended methods as well. Overall, NR suspension can accelerate the aging time of beef carcasses, which reduces the production cost and brings more benefits in beef industry. The present work could also strengthen our view of the temporal expression profile between two suspended methods during post-mortem aging and identify novel biomarkers for meat quality of beef cattle.

## Data Availability Statement

The datasets presented in this study can be found in online repositories. The names of the repositories and accession number(s) can be found below: https://doi.org/10.6084/m9.figshare.16608169, and PRIDE database repository, accession number PXD029806.

## Ethics Statement

The animal study was reviewed and approved by the Animal Care and Ethics Committee of Institute of Animal Science, Chinese Academy of Agricultural Sciences.

## Author Contributions

HL, JL, and YZ: conceived and designed the study. WC: performed the data analysis and wrote the manuscript. KW: performed the beef quality-related experiments. YZ, HZ, and LC: are responsible for the samples collection. All authors contributed to the article and approved the submitted version.

## Funding

This research was supported by Xinjiang Key R&D Program Project (2017B01001-3), China Agriculture Research System of MOF and MARA (CARS-37), Central Public-interest Scientific Institution Basal Research Fund (2020-YWF-YB-02), the Science and Technology Project of Inner Mongolia Autonomous Region (2020GG0210), and the Science and Technology to Revitalize Mongolia Key Project of Inner Mongolia Autonomous Region (Traditional and Molecular Breeding of Beef Cattle, KJXM2020002-01).

## Conflict of Interest

The authors declare that the research was conducted in the absence of any commercial or financial relationships that could be construed as a potential conflict of interest.

## Publisher's Note

All claims expressed in this article are solely those of the authors and do not necessarily represent those of their affiliated organizations, or those of the publisher, the editors and the reviewers. Any product that may be evaluated in this article, or claim that may be made by its manufacturer, is not guaranteed or endorsed by the publisher.

## Supplementary Material

Table S1 The pH, drip loss, cooking loss, color, shear force, and myofibril fragmentation index (MFI) changes between achilles tendon (AT) and neck-arm restraint (NR) suspensions during aging time.

Table S2 Detail information of all the accurately identified proteins by tandem mass tags.

Table S3 The differentially expressed proteins (DEPs) in AT suspension vs. NR suspension at post-mortem days 1, 7, and 14.

Table S4 The DEPs among post-mortem days 1, 7, and 14.

Table S5 The functional annotation of DEPs at post-mortem days 1, 7, and 14 using KOBAS.

Figure S1 **(A–C)** Changes of L^*^, a^*^, and b^*^ between AT and NR suspensions in biceps femoris (BF), longissimus dorsi (LD), and triceps brachii (TB) muscles during post-mortem aging. The lines with green, red, and blue represent BF, LD, and TB, respectively. The solid and dashed line represent AT and NR suspension, respectively. ^*^Symbol means the values are significantly different between AT and NR suspension (*P*-value < 0.05). a–g Means the values are significantly changed during aging (*P*-value < 0.05).

## References

[1] Albino L Prates M Cravo P Silva S Leme P Feitosa G. Hanging the beef carcass by the forequarter to improve tenderness of the Longissimus dorsi and Biceps femoris muscles. Sci Agric. (2005) 82:483–86. 10.1590/S0103-90162005000500013

[2] Dobbs LM Jensen KL Leffew MB English BC Lambert DM Clark CD. Consumer willingness to pay for Tennessee beef. J Food Distribut Res. (2016) 47:38–61. 10.22004/ag.econ.240768

[3] Sørheim O Hildrum KI. Muscle stretching techniques for improving meat tenderness. Trends Food Sci Technol. (2002) 13:127–35. 10.1016/S0924-2244(02)00069-9

[4] Hopkins DL. Tenderizing mechanisms | mechanical. In: Jensen WK, editors. Encyclopedia of Meat Sciences. Oxford: Elsevier (2004). p. 1355–63.

[5] Hostetler RL Link BA Landmann WA Fitzhugh JR. effect of carcass suspension on sarcomere length and shear force of some major bovine muscles. J Food Sci. (1972) 37:132–5. 10.1111/j.1365-2621.1972.tb03402.x

[6] Owens F Gardner B. A review of the impact of feedlot management and nutrition on carcass measurements of feedlot cattle. J Anim Sci. (2000) 77:1–18. 10.2527/jas2000.00218812007700ES0034x

[7] Ahnström ML Hunt MC Lundström K. Effects of pelvic suspension of beef carcasses on quality and physical traits of five muscles from four gender–age groups. Meat Sci. (2012) 90:528–35. 10.1016/j.meatsci.2011.09.003 22077997

[8] Fisher AV Pouros A Wood JD Young-Boong K Sheard PR. Effect of pelvic suspension on three major leg muscles in the pig carcass and implications for ham manufacture. Meat Sci. (2000) 56:127–32. 10.1016/S0309-1740(00)00028-0 22061899

[9] Bouton P Harris P Shorthose R Baxter R. A comparison of the effects of aging, conditioning and skeletal restraint on the tenderness of mutton. J Food Sci. (2006) 38:932–7. 10.1111/j.1365-2621.1973.tb02117.x

[10] Ludwig CJ Claus JR Marriott NG Johnson J Wang H. Skeletal alteration to improve beef longissimus muscle tenderness1. J Anim Sci. (1997) 75:2404–10. 10.2527/1997.7592404x 9303458

[11] Wu W Fu Y Therkildsen M Li X-M Dai R-T. Molecular understanding of meat quality through application of proteomics. Food Rev Int. (2015) 31:13–28. 10.1080/87559129.2014.961073

[12] Zhai C Djimsa BA Prenni JE Woerner DR Belk KE Nair MN. Tandem mass tag labeling to characterize muscle-specific proteome changes in beef during early postmortem period. J Proteomics. (2020) 222:103794. 10.1016/j.jprot.2020.103794 32330628

[13] Kim HJ Sujiwo J Kim HJ Jang A. Effects of dipping chicken breast meat inoculated with listeria monocytogenes in lyophilized scallion, garlic, and kiwi extracts on its physicochemical quality. Food Sci Anim Resour. (2019) 39:418–29. 10.5851/kosfa.2019.e37 31304471 PMC6612791

[14] Gornall AG Bardawill CJ David MM. Determination of serum proteins by means of the biuret reaction. J Biol Chem. (1949) 177:751–66. 10.1016/S0021-9258(18)57021-6 18110453

[15] Xie C Mao X Huang J Ding Y Wu J Dong S. KOBAS 2.0: a web server for annotation and identification of enriched pathways and diseases. Nucleic Acids Res. (2011) 39(Web Server issue):W316–22. 10.1093/nar/gkr483 21715386 PMC3125809

[16] von Mering C Huynen M Jaeggi D Schmidt S Bork P Snel B. STRING: a database of predicted functional associations between proteins. Nucleic Acids Res. (2003) 31:258–61. 10.1093/nar/gkg034 12519996 PMC165481

[17] Shannon P Markiel A Ozier O Baliga NS Wang JT Ramage D. Cytoscape: a software environment for integrated models of biomolecular interaction networks. Genome Res. (2003) 13:2498–504. 10.1101/gr.1239303 14597658 PMC403769

[18] Langberg CW Waldron JA Baker ML Hauer-Jensen M. Significance of overall treatment time for the development of radiation-induced intestinal complications. An experimental study in the rat. Cancer. (1994) 73:2663–8. 10.1002/1097-0142(19940515)73:10<2663::AID-CNCR2820731031>3.0.CO;2-C 8174067

[19] MacLean B Tomazela DM Shulman N Chambers M Finney GL Frewen B. Skyline: an open source document editor for creating and analyzing targeted proteomics experiments. Bioinformatics. (2010) 26:966–8. 10.1093/bioinformatics/btq054 20147306 PMC2844992

[20] Nian Y Allen P Harrison SM Kerry JP. Effect of castration and carcass suspension method on the quality and fatty acid profile of beef from male dairy cattle. J Sci Food Agric. (2018) 98:4339–50. 10.1002/jsfa.8960 29430648

[21] Offer G Trinick J. On the mechanism of water holding in meat: the swelling and shrinking of myofibrils. Meat Sci. (1983) 8:245–81. 10.1016/0309-1740(83)90013-X 22055626

[22] Hou X Liang R Mao Y Zhang Y Niu L Wang R. Effect of suspension method and aging time on meat quality of Chinese fattened cattle M. Longissimus dorsi. Meat Sci. (2014) 96:640–5. 10.1016/j.meatsci.2013.08.026 24056407

[23] Bures D Barton L Bureš D Barton L. Growth performance, carcass traits and meat quality of bulls and heifers slaughtered at different ages. Czech J Anim Sci. (2012) 57:34–43. 10.17221/5482-CJAS

[24] McDonagh MB Fernandez C Oddy VH. Hind-limb protein metabolism and calpain system activity influence post-mortem change in meat quality in lamb. Meat Sci. (1999) 52:9–18. 10.1016/S0309-1740(98)00143-0 22062138

[25] Li K Zhang Y Mao Y Cornforth D Dong P Wang R. Effect of very fast chilling and aging time on ultra-structure and meat quality characteristics of Chinese Yellow cattle M. Longissimus lumborum. Meat Sci. (2012) 92:795–804. 10.1016/j.meatsci.2012.07.003 22857853

[26] Herring HK Cassens RG Rriskey EJ. Further studies on bovine muscle tenderness as influenced by carcass position, sarcomere length, and fiber diameter. J Food Sci. (1965) 30:1049–54. 10.1111/j.1365-2621.1965.tb01885.x

[27] Campos CF Costa TC Rodrigues RT Guimarães SE Moura FH Silva W. Proteomic analysis reveals changes in energy metabolism of skeletal muscle in beef cattle supplemented with vitamin A. J Sci Food Agric. (2020) 100:3536–43. 10.1002/jsfa.10401 32240539

[28] Muniz MMM Fonseca LFS dos Santos Silva DB de Oliveira HR Baldi F Chardulo AL. Identification of novel mRNA isoforms associated with meat tenderness using RNA sequencing data in beef cattle. Meat Sci. (2021) 173:108378. 10.1016/j.meatsci.2020.108378 33248741

[29] Rosa AF Moncau CT Poleti MD Fonseca LD Balieiro JCC Silva SLE. Proteome changes of beef in Nellore cattle with different genotypes for tenderness. Meat Sci. (2018) 138:1–9. 10.1016/j.meatsci.2017.12.006 29289712

[30] D'Alessandro A Marrocco C Rinalducci S Mirasole C Failla S Zolla L. Chianina beef tenderness investigated through integrated Omics. J Proteomics. (2012) 75:4381–98. 10.1016/j.jprot.2012.03.052 22510581

[31] Kwon H-S Lee H-S Ji Y Rubin JS Tomarev SI. Myocilin is a modulator of Wnt signaling. Mol Cell Biol. (2009) 29:2139–54. 10.1128/MCB.01274-08 19188438 PMC2663295

[32] Srinivasan S Avadhani NG. Cytochrome c oxidase dysfunction in oxidative stress. Free Radic Biol Med. (2012) 53:1252–63. 10.1016/j.freeradbiomed.2012.07.021 22841758 PMC3436951

[33] Xiang F Ma S-Y Lv Y-L Zhang D-X Song H-P Huang Y-S. Tumor necrosis factor receptor-associated protein 1 regulates hypoxia-induced apoptosis through a mitochondria-dependent pathway mediated by cytochrome c oxidase subunit II. Burns Trauma. (2019) 7:16. 10.1186/s41038-019-0154-3 31143823 PMC6532166

[34] Laville E Sayd T Morzel M Blinet S Chambon C Lepetit J. Proteome changes during meat aging in tough and tender beef suggest the importance of apoptosis and protein solubility for beef aging and tenderization. J Agric Food Chem. (2009) 57:10755–64. 10.1021/jf901949r 19860418

[35] Polati R Menini M Robotti E Millioni R Marengo E Novelli E. Proteomic changes involved in tenderization of bovine Longissimus dorsi muscle during prolonged ageing. Food Chem. (2012) 135:2052–69. 10.1016/j.foodchem.2012.06.093 22953957

[36] Santos Silva DBD Fonseca LFS Magalhães AFB Muniz MMM Baldi F Ferro JA. Transcriptome profiling of muscle in Nelore cattle phenotypically divergent for the ribeye muscle area. Genomics. (2020) 112:1257–63. 10.1016/j.ygeno.2019.07.012 31351181

[37] Boudon S Henry-Berger J Cassar-Malek I. Aggregation of omic data and secretome prediction enable the discovery of candidate plasma biomarkers for beef tenderness. Int J Mol Sci. (2020) 21:664. 10.3390/ijms21020664 31963926 PMC7013622

[38] Nonneman DJ Shackelford SD King DA Wheeler TL Wiedmann RT Snelling WM. Genome-wide association of meat quality traits and tenderness in swine. J Anim Sci. (2013) 91:4043–50. 10.2527/jas.2013-6255 23942702

[39] Lawson MA. The role of integrin degradation in post-mortem drip loss in pork. Meat Sci. (2004) 68:559–66. 10.1016/j.meatsci.2004.05.019 22062532

[40] Liu X Xiong X Yang J Zhou L Yang B Ai H. Genome-wide association analyses for meat quality traits in Chinese Erhualian pigs and a Western Duroc × (Landrace × Yorkshire) commercial population. Genet Sel Evol. (2015) 47:44. 10.1186/s12711-015-0120-x 25962760 PMC4427942

